# Heterozygous disruption of activin receptor-like kinase 1 is associated with increased arterial pressure in mice

**DOI:** 10.1242/dmm.019695

**Published:** 2015-11-01

**Authors:** María González-Núñez, Adela S. Riolobos, Orlando Castellano, Isabel Fuentes-Calvo, María de los Ángeles Sevilla, Bárbara Oujo, Miguel Pericacho, Ignacio Cruz-Gonzalez, Fernando Pérez-Barriocanal, Peter ten Dijke, Jose M. López-Novoa

**Affiliations:** 1 Departamento de Fisiología y Farmacología, Universidad de Salamanca, Salamanca 37007, Spain; 2 Unidad de Fisiopatología Renal y Cardiovascular, Instituto ‘Reina Sofía’ de Investigación Nefrológica, Salamanca 37007, Spain; 3 Instituto de Investigación Biomédica de Salamanca (IBSAL), Salamanca 37007, Spain; 4 Instituto de Neurociencias de Castilla y León (INCYL), Salamanca 37008, Spain; 5 Departamento de Cardiología, Hospital Universitario de Salamanca, Salamanca 37007, Spain; 6 Department of Molecular Cell Biology, Leiden University Medical Center, Leiden 2333 ZA, The Netherlands

**Keywords:** Activin receptor-like kinase 1, Animal model of human disease, Angiotensin II, Arterial pressure, Catecholamines, Intracerebroventricular injection, Nitric oxide, Sympathetic nervous system

## Abstract

The activin receptor-like kinase 1 (ALK-1) is a type I cell-surface receptor for the transforming growth factor-β (TGF-β) family of proteins. Hypertension is related to TGF-β1, because increased TGF-β1 expression is correlated with an elevation in arterial pressure (AP) and TGF-β expression is upregulated by the renin-angiotensin-aldosterone system. The purpose of this study was to assess the role of ALK-1 in regulation of AP using *Alk1* haploinsufficient mice (*Alk1^+/−^*). We observed that systolic and diastolic AP were significantly higher in *Alk1^+/−^* than in *Alk1^+/+^* mice, and all functional and structural cardiac parameters (echocardiography and electrocardiography) were similar in both groups. *Alk1^+/−^* mice showed alterations in the circadian rhythm of AP, with higher AP than *Alk1^+/+^* mice during most of the light period. Higher AP in *Alk1^+/−^* mice is not a result of a reduction in the NO-dependent vasodilator response or of overactivation of the peripheral renin-angiotensin system. However, intracerebroventricular administration of losartan had a hypotensive effect in *Alk1^+/−^* and not in *Alk1^+/+^* mice. *Alk1^+/−^* mice showed a greater hypotensive response to the β-adrenergic antagonist atenolol and higher concentrations of epinephrine and norepinephrine in plasma than *Alk1^+/+^* mice. The number of brain cholinergic neurons in the anterior basal forebrain was reduced in *Alk1^+/−^* mice. Thus, we concluded that the ALK-1 receptor is involved in the control of AP, and the high AP of *Alk1^+/−^* mice is explained mainly by the sympathetic overactivation shown by these animals, which is probably related to the decreased number of cholinergic neurons.

## Abstract (precis)

**Summary:** Mice haploinsufficient for *Alk1*, a receptor for the TGF-β family, show hypertension, based on overactivation of the sympathetic nervous system and a reduction in the number of cholinergic neurons in the CNS.

## INTRODUCTION

Activin receptor-like kinase 1 (ALK-1) is one of the type I membrane receptors for some of the members of the transforming growth factor-β (TGF-β) family of proteins (Hawinkels et al., 2013). ALK-1 is highly expressed in endothelial cells, but also in many other cell types (Gonzalez-Nunez et al., 2013). ALK-1 participates in the signaling of TGF-β1, TGF-β3, bone morphogenetic protein (BMP) 9 and BMP-10. TGF-β1 and TGF-β3 bind the TGF receptor type II (Pardali and Ten Dijke, 2012). BMP-9 binds preferentially to activin receptor type IIB, whereas BMP-10 binds activin receptor type IIA, activin receptor type IIB or the BMP receptor type II with comparable affinity (Townson et al., 2012). The complex of TGF-β or BMP and the corresponding type II receptor recruits and phosphorylates ALK-1, which becomes activated. Activated ALK-1 in turn phosphorylates the transcription factors Smad1/5/8. Phosphorylated Smads form a heteromeric complex with Smad4, and the complex translocates into the nucleus, where it participates in the activation or repression of the transcription of specific genes (Pardali and Ten Dijke, 2012).

Many of the TGF-β superfamily members are involved in cardiovascular diseases. There are some relationships between hypertension and the ALK-1 ligand TGF-β. Acute administration of TGF-β1 induces vasodilatation and a reduction in arterial pressure (AP; Santibanez et al., 2007). However, increased TGF-β1 expression is associated with an increase in AP (Lavoie et al., 2005). Furthermore, angiotensin II (AngII) and aldosterone, major regulators of AP, increase *TGFβ1* mRNA expression and trigger its transformation to the active form, and AngII also increases the expression of TGF receptor type II mRNA (reviewed by Gordon and Blobe, 2008). In addition, the administration of TGF-β neutralizing antibodies reduces AP in a rat model of hypertension (Lavoie et al., 2005). TGF-β1 also plays a major role in cardiac remodeling by inducing hypertrophy of cardiomyocytes and activation of extracellular matrix production (Bujak and Frangogiannis, 2007; Goumans et al., 2009).

The role of TGF-β superfamily members in atherosclerosis is controversial, but most studies support an inhibitory role for TGF-β in progression of atherosclerosis (Grainger, 2004). However, it has been reported that some BMPs promote progression of atherosclerotic lesions. Increased concentrations of BMP-2, BMP-4 and BMP-6 in these lesions are associated with vascular calcification (Grainger, 2007). Furthermore, the ALK-1 ligand BMP-9 has been reported to be involved in the control of glucose metabolism (Chen et al., 2003) and iron homeostasis (Truksa et al., 2006).

Not only its ligands but also ALK-1 itself is directly implicated in cardiovascular diseases. Mutations of *ACVRL1*, the gene for ALK-1, are associated with the appearance of hereditary hemorrhagic telangiectasia type 2, a vascular dysplasia characterized by telangiectasias in the skin, nose bleeds and arteriovenous malformations in several organs, mainly in the liver (Johnson et al., 1996; Shovlin et al., 1997). In some families with hereditary hemorrhagic telangiectasia type 2 and pulmonary arterial hypertension, variants in the *ACVRL1* gene have been reported. Furthermore, *Alk1* haploinsufficiency in mice is also associated with the appearance of pulmonary arterial hypertension (Jerkic et al., 2011). ALK-1 seems also to be involved in the anti-inflammatory effects of high-density lipoproteins on the vascular endothelium. High-density lipoprotein increases the expression of ALK-1, and this is followed by increases in vascular endothelial growth factor and matrix Gla protein, responsible for the preventive effect of high-density lipoproteins on vascular endothelial inflammation and calcification. These increases are dependent on BMP signaling (Yao et al., 2008). Finally, ALK-1 seems to be able to regulate vascular (Garrido-Martin et al., 2013) and renal fibrosis (Munoz-Felix et al., 2014).

**TRANSLATIONAL IMPACT**

**Clinical issue**

Hypertension, a predominant risk factor for cardiovascular disease, has a complex etiology. Proteins in the transforming growth factor-β family, including TGF-β1, play a major role in the development of hypertension and its complications. TGF-β1 expression is upregulated by the renin-angiotensin-aldosterone system, and this is correlated with an elevation in arterial pressure (AP). Activin receptor-like kinase 1 (ALK-1) is a known cell surface receptor for several members of this family of proteins; however, its possible involvement in hypertension has never been assessed. The purpose of this study was to assess the role of ALK-1 in regulating AP. An *Alk1* haploinsufficient mouse (*Alk1^+/−^*) model and its control (*Alk1^+/+^*) were used to address this question, because *Alk1* knockout mice are not viable.

**Results**

Arterial pressure, heart rate and locomotor activity were measured in *Alk1^+/−^* and control mice using radiotelemetry. Transthoracic echocardiography and telemetric electrocardiogram were also performed. Systolic and diastolic AP were significantly higher in *Alk1^+/−^* than in *Alk1^+/+^* mice. All functional and structural heart parameters were similar in both groups, and electrocardiographic analysis revealed no apparent abnormalities in *Alk1^+/−^* mice. Renal function was also found to be unchanged. Interestingly, *Alk1^+/−^* mice showed alterations in AP circadian rhythm; during the morning (light) period, AP was higher in the haploinsufficient mice than in wild-type control mice. Alterations in the nitric oxide-cGMP vasodilator system or in the peripheral renin-angiotensin system were not detected in *Alk1^+/−^* mice, indicating that the increase in AP was not mediated by these systems.  Nonetheless, intracerebroventricular administration of losartan, an angiotensin receptor antagonist, had a hypotensive effect in *Alk1^+/−^* mice (but not in *Alk1^+/+^* mice). *Alk1^+/−^* mice also demonstrated an increased hypotensive response to the β-adrenergic antagonist atenolol and increased plasma levels of epinephrine and norepinephrine. Confirming a role for the sympathetic nervous system, the authors showed that the number of brain cholinergic neurons is reduced in *Alk1^+/−^* mice.

**Implications and future directions**

This study reports that mice haploinsufficient for *Alk1* present with hypertension and show a marked alteration of the circadian rhythm of AP. The latter finding is reminiscent of the ‘non-dipper’ effect in humans with hypertension, i.e. individuals whose blood pressure does not dip during the night and so persists at a relatively high level throughout a 24 h period. This suggests that *Alk1* haploinsufficient mice could represent a potential model for study of non-dipper hypertension.  In addition to providing new evidence for the involvement of the ALK-1 receptor for cytokines of the TGF-β superfamily in the control of AP, the work also implicates hyperactivation of the sympathetic nervous system as an underlying mechanism for ALK-1-associated hypertension. In line with this, ALK-1 activation is a major regulator of the survival and differentiation of cholinergic neurons. Thus, bone morphogenetic protein-dependent differentiation of brain cholinergic neurons could be a potential therapeutic target for the control of hypertension. Further in-depth studies on the role of ALK-1 in vascular homeostasis are needed to determine how to target this signaling pathway in a specific way.

An important role for ALK-1 in vascular homeostasis is thus evident. The purpose of this study, therefore, was to assess the role of ALK-1 in regulation of AP, using a previously described mouse model of *Alk1* haploinsufficiency (*Alk1*^+/−^), because *Alk1* knockout mice are embryonic lethal (Oh et al., 2000). Our data provide the first demonstration of the involvement of the ALK-1 signaling pathway in the control of AP.

## RESULTS

### Phenotypic characterization of *Alk1* heterozygous mice

To analyze the expression of ALK-1, the mRNA and protein levels were measured in lung and kidneys from *Alk1*^+/−^ and *Alk1^+/+^* adult mice, because ALK-1 is mainly expressed in vascularized tissues (Gonzalez-Nunez et al., 2013). Our results show that ALK-1 protein (Fig. S1A) and mRNA content (Fig. S1B) in *Alk1*^+/−^ mice is about half of that found in lungs and kidneys from *Alk1^+/+^* littermates. These data corroborate that *Alk1*^+/−^ mice are haploinsufficient for *Alk1*. Furthermore, we also analyzed other important components of the ALK-1 signaling pathway, such as the receptors ALK-5 and Endoglin, and we observed that their levels were not different between *Alk1*^+/−^ and *Alk1^+/+^* mice (Fig. S1A,B).

### *Alk1*^+/−^ mice show increased arterial pressure with normal cardiac and renal function

**Fig. 1.**
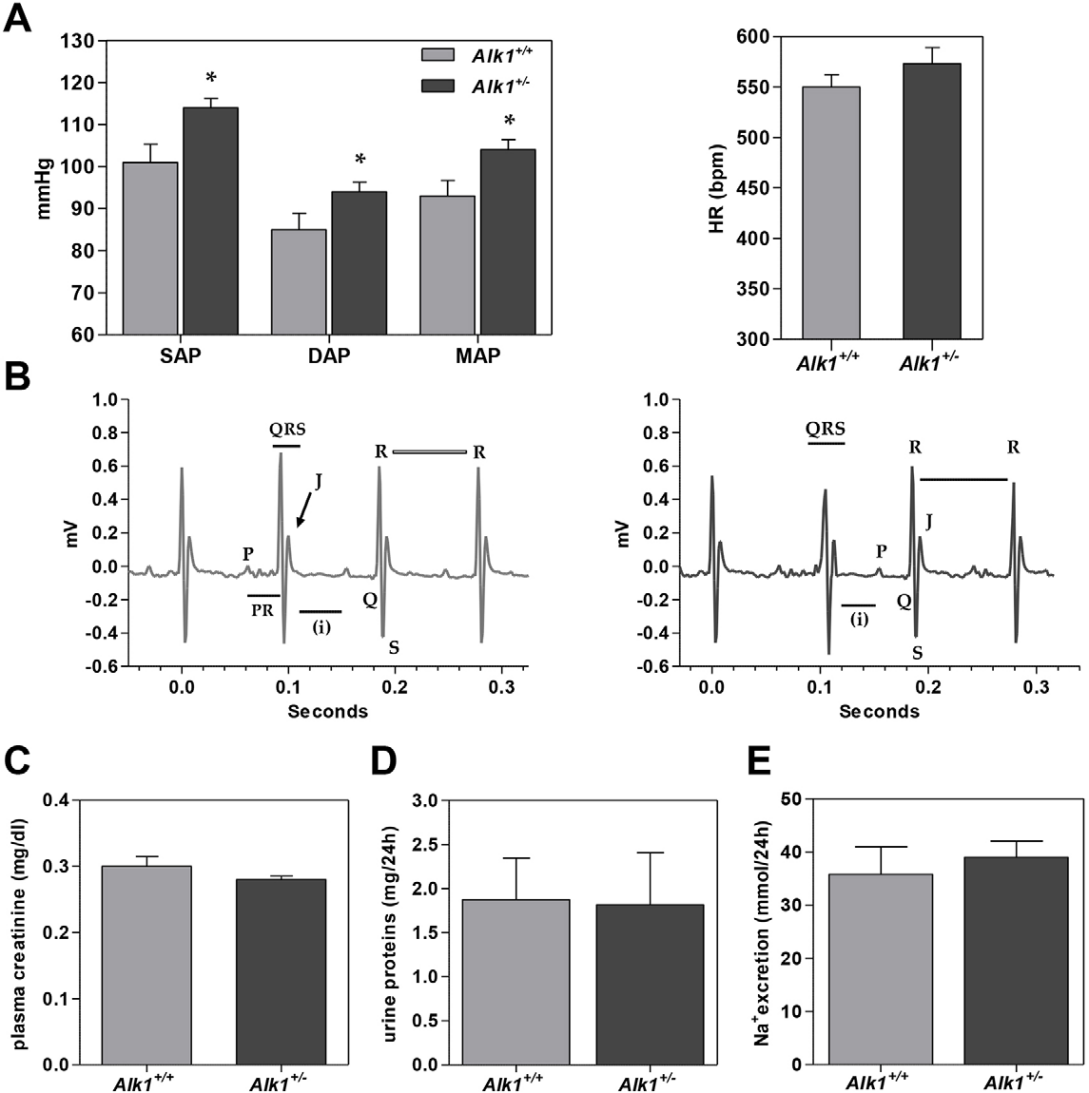
**Hemodynamic and renal function analysis of *Alk1^+/−^* mice.** (A) Basal radiotelemetric measurements of systolic (SAP), diastolic (DAP) and mean arterial pressure (MAP) and heart rate (HR) of male *Alk1^+/+^* and *Alk1^+/−^* mice. Results correspond to mean values of control (*n*=8) and heterozygous mice (*n*=8). (B) Representative images of a basal radiotelemetric recordings of electrocardiogram in *Alk1^+/+^* (left panel, *n*=6) and *Alk1^+/−^* mice (right panel, *n*=6). The images show a 0.3 s record with a typical P wave, QRS complex, mouse characteristic J wave and an isoelectric zone (i). (C-E) Plasma creatinine (C; *Alk1^+/+^*, *n*=13; *Alk1^+/−^*, *n*=15), urinary excretion of proteins (D; *Alk1^+/+^*, *n*=6; *Alk1^+/−^*, *n*=6) and urinary excretion of Na^+^ (E; *Alk1^+/+^*, *n*=6; *Alk1^+/−^*, *n*=6) in basal conditions. Data are shown as mean±s.e.m. *P<0.05 *Alk1^+/−^* versus *Alk1^+/+^*.

Measurements of AP by the tail-cuff method (data not shown) and by telemetry showed higher systolic and diastolic AP in *Alk1*^+/−^ than in *Alk1^+/+^* mice with no significant differences in heart rate (HR; Fig. 1A). As increased cardiac function is a possible cause of increased AP, measurement of cardiac function and structure was performed by echocardiography in *Alk1*^+/−^ and *Alk1^+/+^* mice. All functional and structural parameters, either directly measured or calculated, were similar in both groups of animals (Table S1).

Electrocardiographic (ECG) analysis revealed no apparent abnormalities in *Alk1^+/+^* or *Alk1^+/−^* mice, because 24 h continuous telemetry ECG recordings demonstrated that wave intensity, RR interval, PQ interval, QRS duration and QT interval were similar in *Alk1*^+/−^ and *Alk1^+/+^* mice (Fig. 1B). No differences were observed in the ECG pattern at different moments of the day, except for the circadian changes in HR. The ventricular wall histology did not show differences between the two types of mice (Fig. S2A).

As the kidney plays a key role in the regulation of AP, we also wanted to rule out a defect in kidney function in mice heterozygous for *Alk1* as a cause of increased AP. To assess renal function, plasma creatinine (Fig. 1C), creatinine clearance (data not shown), urinary protein excretion (Fig. 1D) and urinary sodium excretion (Fig. 1E) were measured, and no significant differences were found between *Alk1^+/+^* and *Alk1^+/−^* mice. The microscopic structure of the kidneys (hematoxylin and eosin or Masson's trichrome staining) was similar in both groups (Fig. S2B).

### *Alk1*^+/−^ mice show alterations in arterial pressure circadian rhythm

**Fig. 2.**
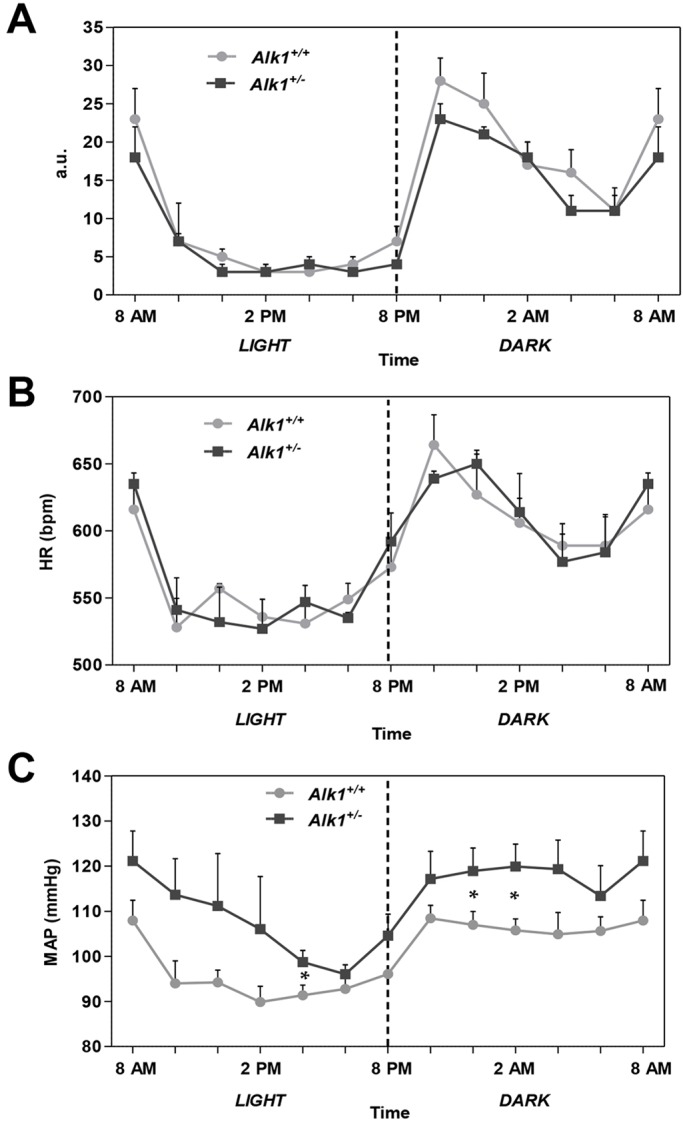
**Circadian rhythms.** (A-C) Circadian  rhythm of activity (A), heart rate (HR; B) and mean arterial pressure (MAP; C). Results correspond to records during the 24 h obtained by telemetry in *Alk1^+/+^* (*n*=6) and *Alk1^+/−^* mice (*n*=7) and are divided into 2 h sections. Mice had complete freedom of movement, and the conditions of light-dark, feed and temperature were kept constant throughout the experiment. a.u., arbitrary units; 0, periods of low activity or total inactivity. *P<0.05 *Alk1^+/−^* versus *Alk1^+/+^*.

As AP can be affected by sleep-wake circadian cycles and locomotor activity cycles, AP and locomotor activity were continuously assessed by telemetry for a 24 h period and analyzed each 2 h. The periods of activity and inactivity were similar in both groups of animals (Fig. 2A). The areas that corresponded to higher activity were around 08.00 h, preceding ‘lights on’, and between 22.00 and midnight, after ‘lights off’, remaining relatively high during the dark period. There was a marked decreased activity between 11.00 and 19.00 h (light period), bordering zero activity.

There was a similar oscillation in HR in the two groups of animals (Fig. 2B), with a profile that fits well to motor activity, and without significant differences between *Alk1^+/+^* and *Alk1^+/−^* mice in any of the periods studied.

AP also has a circadian rhythm, with maximal values around 08.00 h, preceding ‘lights on’ and relatively high values throughout the dark period from 20.00 to 08.00 h. The lower values of AP in *Alk1^+/+^* mice were observed during the light period (from 10.00 to 20.00 h), with an abrupt fall in AP from 08.00 to 10.00 h. However, in *Alk1^+/−^* mice, this reduction in AP was less pronounced, and these mice maintained higher AP than *Alk1^+/+^* mice during most of the light period, with the maximal differences during the morning, which is the time when most of the experiments with the mice were performed (Fig. 2C). *Alk1^+/−^* mice also had significantly higher AP around midnight compared with *Alk1^+/+^* mice (Fig. 2C).

### *Alk1*^+/−^ mice show no impairment in the nitric oxide-cGMP vasodilator system

As we found no alterations in either cardiac or renal function, we assessed the possibility that the increase in AP of heterozygous *Alk1* mice could be caused by an alteration in the peripheral vascular tone. In order to test this possibility, we first assessed the endothelium-dependent vasodilator response. Acute administration of acetylcholine (ACh) induced a similar reduction in AP in heterozygous and *Alk1^+/+^* mice (Fig. S3A). Acute administration of sodium nitroprusside (SNP) also induced a significant reduction in systolic AP in *Alk1^+/−^* and *Alk1^+/+^* mice, without a different effect between the groups (Fig. S3B).

Next, we determined the response of AP to the blockade of NO synthesis. Acute administration of the nitric oxide synthase (NOS) inhibitor L-NAME induced a sustained and similar hypertensive response in both groups of animals (Fig. S3C). Chronic administration during 8 weeks induced a significant time-dependent increase in AP and HR that was similar in both groups of mice (Fig. S3D).

In order to investigate the effect of both vasodilators on arterial relaxation further, isolated aortic rings from the two groups were used. Aortic rings from *Alk1^+/+^* and *Alk1*^+/−^ mice were first stimulated to 80-90% of maximal contraction with phenylephrine (PE), and increasing concentrations of ACh induced a marked and dose-dependent relaxation that was similar in aortic rings from both groups of mice (Fig. S3E). By contrast, although maximal relaxation of the aortic rings induced by the NO donor SNP was identical between genotypes, the concentration-response curve for SNP was shifted to the left in vessels from *Alk1^+/−^* mice (Fig. S3F).

The levels of the endothelial-type constitutive NOS (eNOS) in aorta and kidney were found to be higher in *Alk1^+/−^* mice, as determined by western blot (Fig. S4A and B), but this difference was statistically significant only in aorta. The urinary concentration of nitrites, stable-end products of NO metabolism, was not different in *Alk1^+/−^* and *Alk1^+/+^* mice (Fig. S4C). Levels of cyclooxygenase-2 in aorta and kidney were found to be similar in *Alk1^+/−^* and *Alk1^+/+^* mice (Fig. S4A,B).

### *Alk1*^+/−^ mice show no overactivation of the peripheral renin-angiotensin system

The renin-angiotensin system plays a major role in the control of AP; therefore, we aimed to assess the function of this system in *Alk1*^+/−^ mice.

**Fig. 3.**
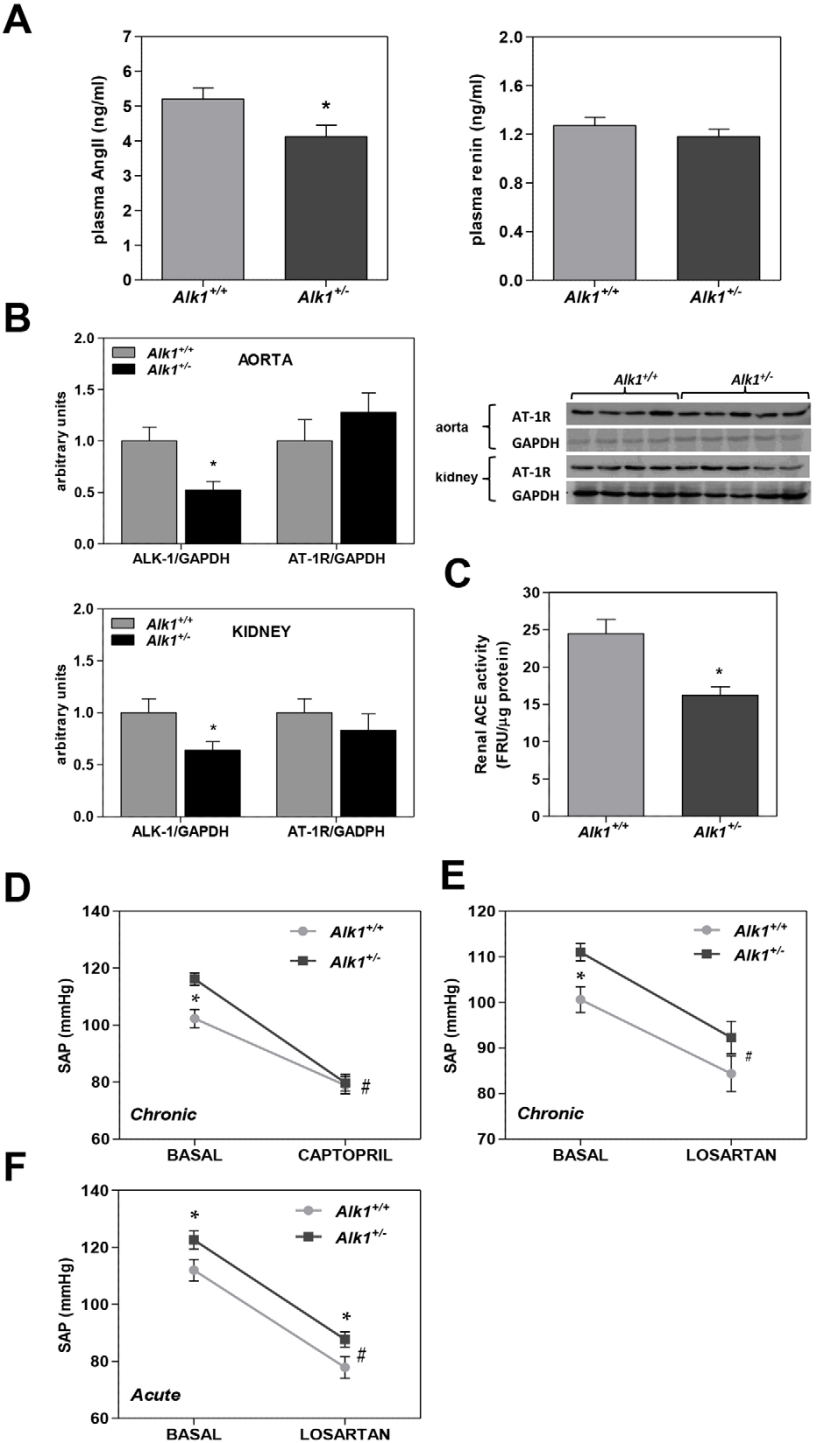
**Assessment of renin-angiotensin system activity.** (A) Plasma levels of angiotensin II (*Alk1^+/+^*, *n*=5; *Alk1^+/−^*, *n*=5) and renin (*Alk1^+/+^*, *n*=12; *Alk1^+/−^*, *n*=14). (B) Western blot analysis of ALK-1 and AT-1R protein in aorta and kidney (*Alk1^+/+^*, *n*=4; *Alk1^+/−^*, *n*=5). (C) ACE activity in renal tissue of *Alk1^+/+^* (*n*=6) and *Alk1^+/−^* mice (*n*=6). (D) Chronic effects of captopril on SAP in *Alk1^+/−^* (*n*=6) and *Alk1*^+/+^ mice (*n*=6). (E) Chronic effects of losartan on SAP in *Alk1^+/+^* (*n*=6) and *Alk1*^+/+^ mice (*n*=6). (F) Acute effect of losartan on SAP in *Alk1^+/+^* (*n*=8) and *Alk1*^+/+^ mice (*n*=13). Chronic effects were assessed by the tail-cuff method and the acute effect on blood pressure was assessed by telemetry. ACE, angiotensin II-converting enzyme; AngII, angiotensin II; AT-1R, AT-1 receptor; FRU, fluorescence relative units; SAP, systolic arterial pressure. Data are means±s.e.m. *P<0.05, *Alk1^+/−^* versus *Alk1^+/+^*; ^#^*P*<0.05, treated *Alk1^+/−^* versus treated *Alk1^+/+^*.

Plasma renin levels were not statistically different between control and *Alk1^+/−^* mice, whereas AngII levels were slightly but significantly lower in *Alk1^+/−^* mice (Fig. 3A). Western blot analysis showed a similar content of the AngII type 1 receptor (AT-1R) in aorta and kidney in both groups, as shown in Fig. 3B. Furthermore, renal tissue angiotensin II-converting enzyme activity was lower in *Alk1^+/−^* than in *Alk1^+/+^* mice (Fig. 3C).

Administration of captopril for 4 days decreased systolic arterial pressure (SAP) in *Alk1^+/−^* mice (by ∼35 mmHg) but produced less change in *Alk1*^+/+^ mice (∼26 mmHg), as assessed by the tail-cuff method (Fig. 3D). Administration of the AT1 antagonist losartan for 4 days was similarly effective in lowering SAP (assessed by tail-cuff method) in *Alk1^+/−^* and *Alk1*^+/+^ mice (Fig. 3E). Acute administration of losartan induced a similar hypotensive effect in AP in *Alk1^+/−^* and *Alk1*^+/+^ mice (Fig. 3F).

The acute i.p. administration of AngII in conscious mice had the same effect on AP measured by telemetry in both groups for all doses used, with an increase in SAP of ∼60 mmHg at the highest dose (0.8 mg/kg body weight; Fig. S5A). After i.v. administration, the increase in AP measured by direct cannulation of the carotid artery in anesthetized animals was significantly lower in *Alk1*^+/−^ than in *Alk1^+/+^* mice at a dose of 0.15 mg/kg body weight but was similar with 0.30 mg/kg body weight (Fig. S5B).

To evaluate the effect of administration of AngII exclusively on vascular resistance, we used a model of hindquarter perfusion in mice. The change in vascular resistance in response to AngII was not statistically different between the two groups of animals at the two doses used (Fig. S5C). Furthermore, the contractile response to AngII was less in *Alk1^+/−^* than in *Alk1*^+/+^ aortic rings, although this difference was not statistically significant (Fig. S5D).

### *Alk1*^+/−^ mice show sympathetic nervous system overactivation

**Fig. 4.**
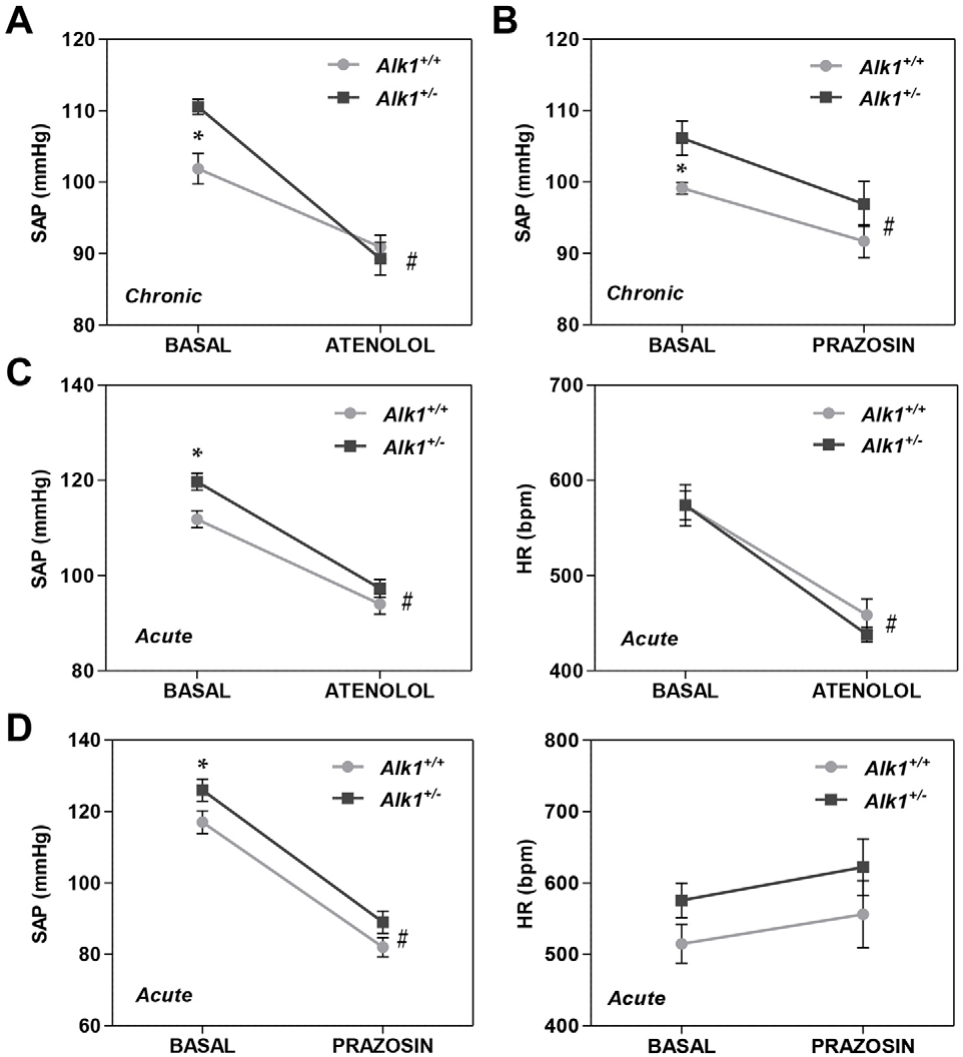
**Pressure responses to agonists or antagonists of the sympathetic nervous system.** (A,B) Effects of chronic administration of atenolol (A) and prazosin (B) on SAP in *Alk1^+/+^* (*n*=5) and *Alk1^+/−^* mice (*n*=5). Chronic effects on blood pressure were assessed by the tail-cuff method. (C) Effects of acute administration of atenolol on SAP and HR in *Alk1^+/+^* (*n*=12) and *Alk1^+/−^* mice (*n*=15). (D) Effects of acute administration of prazosin on SAP and HR in *Alk1^+/+^* (*n*=11) and *Alk1^+/−^* mice (*n*=12). Acute effects on blood pressure were assessed by telemetry. HR, heart rate; SAP, systolic arterial pressure. *P<0.05 *Alk1^+/−^* versus *Alk1^+/+^*; ^#^*P*<0.05 treatment versus basal.

**Fig. 5.**
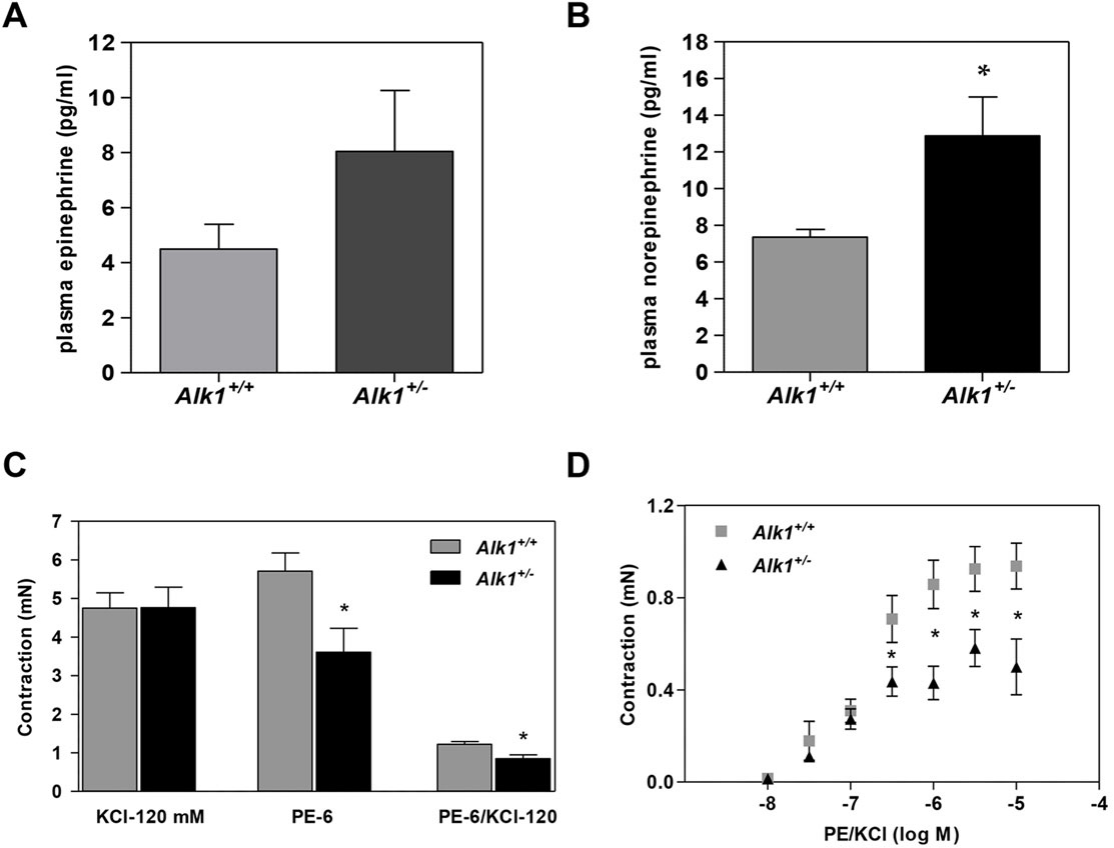
**Plasma levels of epinephrine and norepinephrine and contractile responses to phenylephrine.** (A,B) Plasma levels of epinephrine (A) and norepinephrine (B) in *Alk1^+/−^* (*n*=8) and *Alk1^+/+^* mice (*n*=6). (C) Maximal contractile response to KCl (120 mM) and (10^−6^ M) of aortic rings from *Alk1^+/+^* (*n*=12) and *Alk1^+/−^* mice (*n*=12). (D) Dose-dependent contractile effect of PE (10^−8^-10^−5^ M). PE, phenylephrine. Data are means±s.e.m. *P<0.05 *Alk1^+/−^* versus *Alk1^+/+^*.

Our next aim was to assess the role of overactivation of the sympathetic nervous system in the high AP shown by *Alk1^+/−^* mice. The administration of atenolol, a β-adrenergic receptor antagonist, for 4 days resulted in a marked decrease in AP in *Alk1^+/−^* mice, and this decrease was markedly lower in *Alk1^+/+^* mice (Fig. 4A). Administration of the α-adrenergic receptor antagonist prazosin for 4 days also induced a decrease in AP that was slightly more pronounced in *Alk1^+/−^* mice (Fig. 4B). The hypotensive response to acute atenolol administration was greater in *Alk1^+/−^* than in *Alk1^+/+^* mice, and a similar response was observed in HR (Fig. 4C). However, the hypotensive response to acute administration of prazosin was similar in *Alk1^+/−^* and *Alk1^+/+^* mice, and no significant effects were observed on HR (Fig. 4D). Plasma concentrations of epinephrine and norepinephrine were higher in *Alk1^+/−^* than in *Alk1^+/+^* mice (Fig. 5A,B, respectively).

Aortic rings from *Alk1^+/−^* and *Alk1^+/+^* mice showed similar contraction in response to KCl (Fig. 5C). The response to increasing doses of PE (from 10^−8^ to 10^−5^ M) was significantly lower in *Alk1^+/−^* than in *Alk1^+/+^* mice, even if this was corrected by KCl-induced contraction (Fig. 5C,D).

### *Alk1*^+/−^ mice show increased activity of the renin-angiotensin system in the central nervous system

**Fig. 6.**
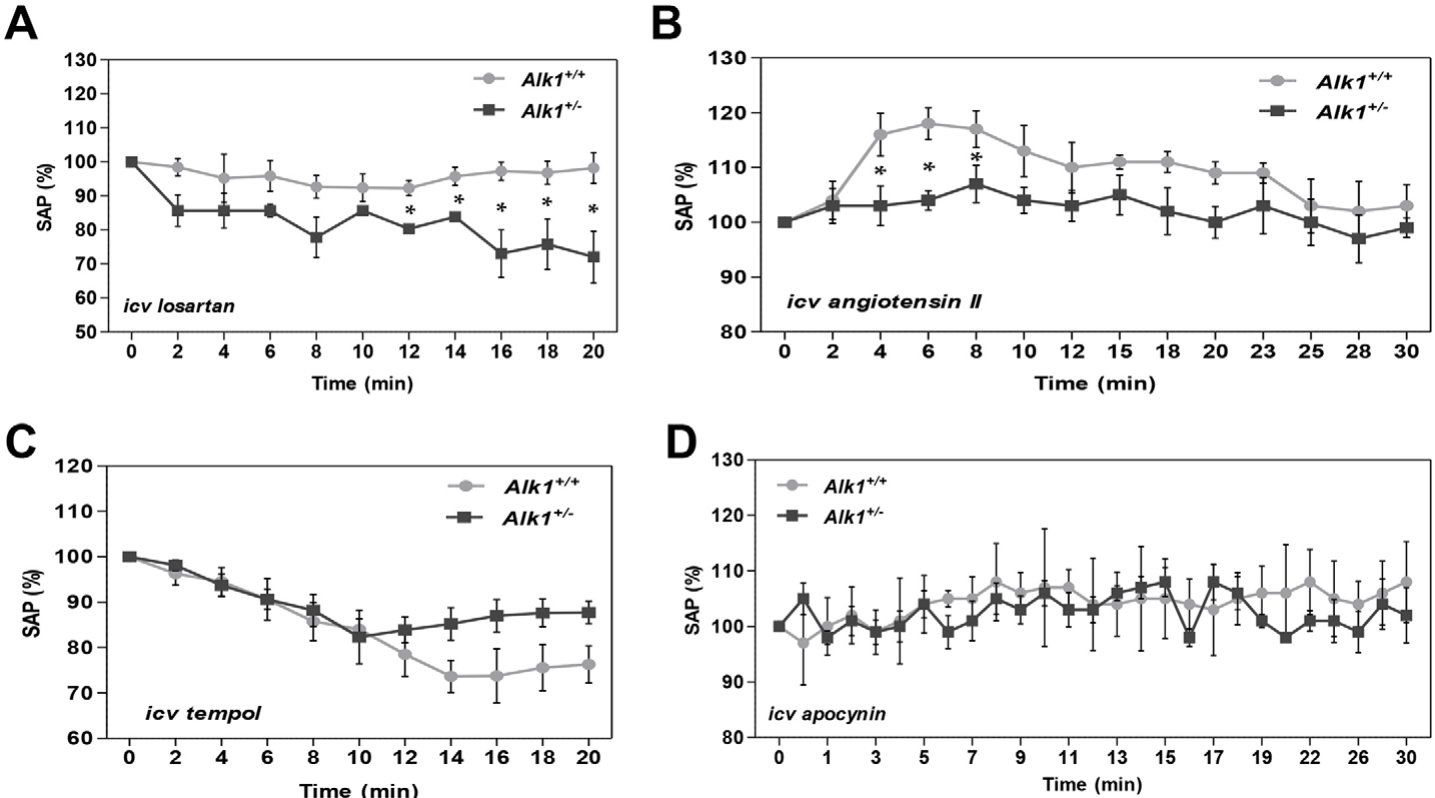
**Changes in systolic arterial pressure in response to intracerebroventricular injections.** (A) Losartan (*Alk1^+/+^*, *n*=4; *Alk1^+/−^*, *n*=4). (B) Angiotensin II (*Alk1^+/+^*, *n*=4; *Alk1^+/−^*, *n*=5). (C) Tempol (*Alk1^+/+^*, *n*=4; *Alk1^+/−^*, *n*=6). (D) Apocynin (*Alk1^+/+^*, *n*=4; *Alk1^+/−^*, *n*=4). i.c.v., intracerebroventricular. Data are means±s.e.m. *P<0.05 *Alk1^+/−^* versus *Alk1^+/+^*.

Given that AngII plays a major role in the central nervous system (CNS) in sympathetic activation and increased AP, we next assessed this system in *Alk1^+/−^* mice. Intracerebroventricular (i.c.v.) administration of the AngII receptor antagonist losartan induced a significant decrease in AP in *Alk1^+/−^* (∼20 mmHg) but not in *Alk1^+/+^* mice (Fig. 6A). In *Alk1^+/+^* mice i.c.v. administration of AngII caused an increase in AP 6 min after injection of between 15 and 20 mmHg. The response in heterozygous mice was less pronounced and more attenuated, as it increased only 5-8 mmHg 8 min after injection (Fig. 6B).

Intracerebroventricular administration of the superoxide dismutase mimetic tempol resulted in a decrease in AP that was not statistically different between the two groups (Fig. 6C). Intracerebroventricular administration of the NAD(P)H oxidase inhibitor apocynin had no effect on the AP either in control mice or in *Alk1* heterozygous mice (Fig. 6D). These results led us to discard the possibility that superoxide anion production in excess in the brain of *Alk1^+/−^* mice was responsible for the higher AP observed in them, either directly or through central renin-angiotensin system hyperactivation mediated by an increase in reactive oxygen species production.

### *Alk1*^+/−^ mice have low numbers of cholinergic neurons in the central nervous system

**Fig. 7.**
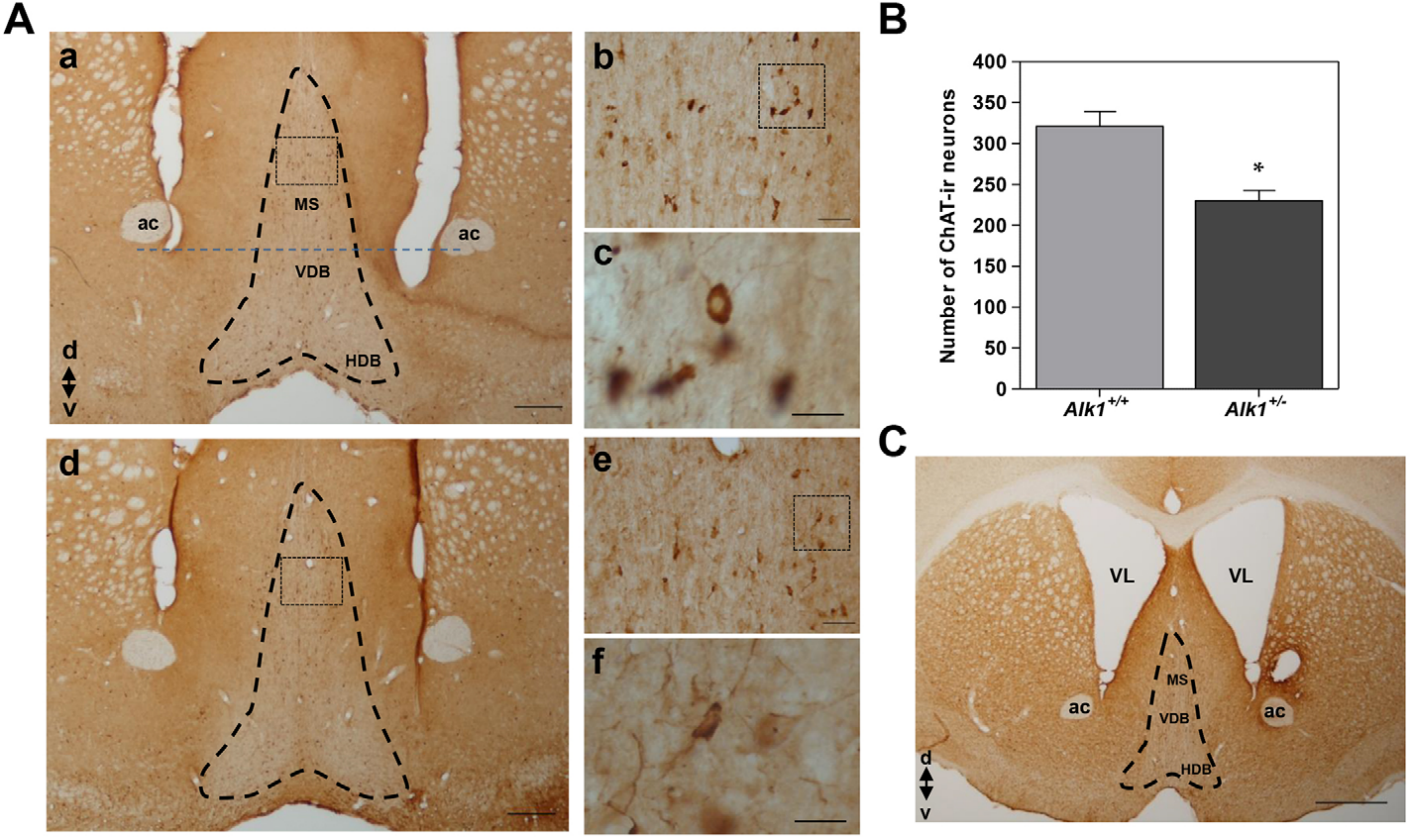
**Photomicrographs of immunostaining against the ChAT enzyme in the basal forebrain.** (A) Representative images of an *Alk1^+/+^* mouse (a-c) and an *Alk1^+/−^* mouse (d-f)*.* The region above the blue dashed line and within the black dashed line delimits the area where the quantitative study was conducted, involving the MS. Scale bars: 500 μm (a,d), 200 μm (b,e) and 50 μm (c,f). The images correspond to 0.86 mm rostral to bregma. (B) Quantification of immunoreactive neurons to the antiserum against ChAT in sections from *Alk1^+/+^* (*n*=3) and *Alk1^+/−^* mice (*n*=3). (C) Representative basal forebrain photomicrograph of an *Alk1^+/−^* mouse*.* There is a marked dilatation of the lateral ventricles, with a reduction in the cholinergic region of the basal forebrain (the area within the dashed line). Scale bar: 1 mm. ac, anterior commissure; ChAT, choline acetyl transferase; ChAT-ir, choline acetyl transferase immunoreactive; d, dorsal; HDB, horizontal limb of diagonal band of Broca; MS, medial septum; v, ventral; VDB, vertical limb of diagonal band of Broca; VL, lateral ventricle. The image corresponds to 0.86 rostral to bregma. *P<0.05 *Alk1^+/−^* versus *Alk1^+/+^*.

As there is a strong relationship between the TGF-β1/ALK-1 system and the maturation of cholinergic neurons, as recently described(Lopez-Coviella et al., 2000; Schnitzler et al., 2010), we considered the possibility that *Alk1^+/−^* mice could have some kind of difference in the cholinergic population in the CNS. Thus, we assessed the presence of choline acetyl transferase-immunoreactive (ChAT-ir) neurons in the anterior basal forebrain from *Alk1^+/+^* and *Alk1^+/−^* mice (Fig. 7Aa,d). The qualitative assessment of the expression of the choline acetyl transferase (ChAT) enzyme in neurons from the basal forebrain showed differences between the two groups. Most of the neurons in *Alk1^+/+^* mice showed great homogeneity and intensity of the immunocytochemical staining, which occupied almost the entire neuronal soma except for the nuclear region (Fig. 7Ab,c), whereas in *Alk1^+/−^* mice the neuronal staining showed greater heterogeneity and much less intensity, with a tendency to be located toward the periphery of the neuronal soma (Fig. 7Ae,f). The quantitative study of the cholinergic population of the medial septum (the area more accessible and better studied) showed a significant reduction in the number of ChAT-ir neurons in *Alk1^+/−^* animals compared with their controls. The mean number of ChAT-ir neurons observed in *Alk1^+/−^* mice was significantly lower than in *Alk1^+/+^* mice (Fig. 7B). Furthermore, some *Alk1^+/−^* mice showed a significant ventricular dilatation (Fig. 7C), a distortion of brain anatomy, which was not observed in any of the *Alk1^+/+^* mice studied. The animals showing this alteration were not used in the quantitative study reported here.

## DISCUSSION

In the present experiments, AP has been measured in *Alk1*^+/−^ and in *Alk1^+/+^* mice by both tail-cuff and radiotelemetry methods. We have used both methods to measure AP because movement restriction necessary for tail-cuff measurement can modify vasoactive responses, especially when the sympathetic nervous system is involved, and because acute (minutes) effects can be difficult to assess by the tail-cuff method. Besides, both acute and prolonged effects of the vasoactive substances have been recorded because the acute and the long-standing consequences of inhibiting or stimulating these regulatory pathways can be different (Emanueli et al., 1997). Measurements of AP by the tail-cuff method and by telemetry showed consistently higher SAP in *Alk1*^+/−^ than in *Alk1^+/+^* mice, with no significant differences in HR. It should be noted that arterial hypertension has not been reported as a common sign in individuals with hereditary hemorrhagic telangiectasia type 2, a fact that can be explained by the multigenetic causes of hypertension in humans (Mongeau, 1987). However, both individuals with hereditary hemorrhagic telangiectasia type 2 and *Alk1* haploinsufficient mice show pulmonary hypertension (Jerkic et al., 2011).

As increased cardiac function is a possible cause of increased AP, measurement of cardiac function was performed by echocardiography. All echocardiographic functional or structural parameters assessed were similar in both groups of animals. Furthermore, no differences between *Alk1^+/−^* and *Alk1^+/+^* mice were observed in the ECG pattern. Moreover, the ventricular wall histology did not show differences between the two types of mice. These results suggest that increased AP in *Alk1*^+/−^ mice cannot be attributed to alterations in cardiac function or structure.

As the kidney plays a key role in the regulation of AP, we also wanted to rule out a defect in kidney function in mice heterozygous for *Alk1* as a cause of increased AP. No significant differences were found in plasma creatinine, creatinine clearance and urinary sodium excretion between *Alk1^+/+^* and *Alk1^+/−^* mice. In addition, kidney structure was similar in both types of mice. Therefore, it does not seem that the increased AP in *Alk1^+/+^* mice is based on alterations in renal function or structure.

The lack of noteworthy alterations in cardiac and renal function and structure in *Alk1^+/−^* mice in the presence of hypertension can probably be explained by the short duration of the hypertension, because the parameters of kidney and cardiac function assessed in the present study typically do not exhibit abnormalities in hypertension until end-organ complications ensue. However, our results do not rule out minor abnormalities in the heart and/or kidney given that molecular and/or cellular analyses have not been done.

As AP can be affected by sleep-wake circadian cycles and locomotor activity cycles, AP and locomotor activity were also assessed continuously by telemetry. We have found that the periods of activity and inactivity were similar in both groups of animals throughout the day, with peaks around the times of ‘light on’ and ‘light off’ and minimal values during the light period, as previously reported for the C57Bl/6 mice strain (Hoit et al., 2002). Therefore, differences in AP were not a result of ongoing maintenance of increased locomotor activity in *Alk1* heterozygous mice, because the profile of changes in locomotor activity was similar in both types of mice. However, the circadian rhythm of AP does not follow the same profile in the two groups of mice, because heterozygous *Alk1^+/−^* mice have a higher AP than *Alk1^+/+^* mice during the morning (light period). In *Alk1^+/+^* mice, there is an abrupt reduction in AP after the lights are turned on (in parallel with the decrease in locomotor activity), whereas in the *Alk1^+/−^* mice this decrease is less pronounced.

This lack of decrease in AP in the light periods in *Alk1^+/−^* mice is reminiscent of ‘non-dipper’ hypertension in humans. It should be noted that loss or blunting of the nocturnal dip is associated with more severe end-organ damage and increased risk of cardiovascular events, and ‘non-dipper’ hypertensive individuals frequently belong to high- or very high-risk categories (de la Sierra et al., 2010). Thus, we can suggest that the *Alk1^+/−^* mice can be considered an experimental model of ‘non-dipper’ hypertension. Nonetheless, to demonstrate this suggestion, we should perform studies in older animals, in which the consequences of hypertension could be more evident.

We then examined the endothelium-dependent vasodilatory responses in *Alk1^+/+^* and *Alk1*^+/−^ animals by measuring the response to NO-dependent vasodilators. The interest in studying this system comes from the fact that the presence of the TGF-β co-receptor endoglin increases signaling through the ALK-1/Smad1 pathway (Lebrin et al., 2004), and previous studies reported that endoglin haploinsufficiency is associated with reduced NO production and eNOS expression and activity (Jerkic et al., 2004; Toporsian et al., 2005). Acute administration of ACh or SNP induced a significantly fall in SAP in *Alk1^+/−^* and *Alk1^+/+^* mice, with no significant differences in the extent of reduction in SAP between both groups of mice. Acute or chronic administration of the NOS inhibitor L-NAME induced a similar hypertensive response in both groups of animals. Furthermore, we found that in aortic rings precontracted with PE, ACh induced a dose-dependent relaxation that was similar in both groups of animals, whereas rings from *Alk1^+/−^* mice were more sensitive to relaxation induced by SNP, indicating that there is no lack of ability of the smooth muscle of *Alk1^+/−^* mice to respond to NO. We also investigated possible changes in endothelial-type constitutive eNOS and NO synthesis in both groups of animals, and we found higher levels of eNOS in aortas and kidneys from *Alk1^+/−^* mice but similar urinary excretion of nitrites, the stable end-products of NO metabolism. Thus, the higher AP of *Alk1^+/−^* mice is not a result of reduced NO-mediated vasodilator activity, whereas the levels of cyclooxygenase-2 were also similar in *Alk1^+/−^* and *Alk1^+/+^* mice.

We next assessed the possible role of renin-angiotensin system activation in the hypertension shown by *Alk1^+/−^* mice. All the parameters studied and the response to AngII and inhibitors or blockers of this system were similar in *Alk1^+/−^* and *Alk1^+/+^* mice, with the exception of plasma AngII levels, which were slightly but significantly lower in *Alk1^+/−^* than in *Alk1^+/+^* mice. Angiotensin II-converting enzyme activity in kidney was lower in *Alk1^+/−^* mice, although this effect probably does not explain the decreased circulating levels of AngII. As levels of AngII regulate the expression of AT-1Rs, we also analyzed by western blot the AT-1R content in aorta and kidney, and this revealed no differences between the groups. Thus, changes in the renin-angiotensin system do not explain the increased AP shown by *Alk1^+/−^* mice.

Sympathetic nervous system activity plays a major role in the etiology of hypertension (Grassi, 2010). Thus, we studied the possible role of sympathetic nervous system overactivation in the hypertension shown by *Alk1^+/−^* mice. We observed that plasma epinephrine and norepinephrine concentrations were higher in *Alk1^+/−^* than in *Alk1^+/+^* mice. Furthermore, the administration of the β-adrenergic receptor antagonist atenolol induced a more intense decrease in AP in *Alk1^+/−^* mice than in *Alk1^+/+^* mice, an effect that was similar but less intense for the α-adrenergic receptor antagonist prazosin. These data suggest that sympathetic activation plays an important role in the generation of the increased AP in *Alk1^+/−^* mice.

In order to investigate this mechanism, we assessed the effect of PE, a selective α_1_-adrenergic agonist, in the contractile force developed by aortic rings, and we observed that contraction was significantly less in aortic rings from *Alk1^+/−^* mice than in those from *Alk1^+/+^* mice. It is well established that α_1_-adrenergic receptors are less phosphorylated, desensitized and internalized when they are chronically exposed to norepinephrine or epinephrine (Akinaga et al., 2013). Therefore, high levels of circulating norepinephrine could cause a reduction in the number of α_1_-receptors, which explains the effect of PE in aortic rings of *Alk1^+/−^* mice. Furthermore, it was also reported that prolonged exposure to catecholamines increases sensitivity to smooth muscle relaxation induced by SNP because of changes in the cGMP system capacity (Hu et al., 1992), which could also explain why aortic rings from *Alk1^+/−^* mice showed a higher percentage of relaxation to SNP.

As AngII in the CNS plays a major role in sympathetic activation and increased AP (Arnold et al., 2013), we next assessed this system in our mouse model. Administration of the AngII receptor antagonist losartan into the lateral cerebral ventricle induced a significant decrease in AP in *Alk1^+/−^* but not in *Alk1^+/+^* mice. This effect led us to think about the possibility of the existence of a higher central renin-angiotensin system overactivity in *Alk1^+/−^* mice. Intracerebroventricular administration of AngII caused a smaller and delayed increase in AP in *Alk1^+/−^* than in *Alk1^+/+^* mice. Thus, *Alk1^+/−^* mice are more sensitive to the hypotensive effects of i.c.v. losartan and less sensitive to the hypertensive effects of i.c.v. AngII, suggesting increased levels of AngII in the CNS of *Alk1^+/−^* mice. Therefore, our results suggest that, although the peripheral renin-angiotensin system seems to have no role in generation of the higher arterial pressure in *Alk1^+/−^* mice, it could have a role at the central level.

It has been reported that increased oxidative stress in several areas of the brain is associated with hypertension and sympathetic overactivation in several animal models (Kishi et al., 2012; Nishihara et al., 2012; Oliveira-Sales et al., 2010). To assess whether the increased AP of *Alk1^+/−^* mice could be mediated by increased production of NADPH-dependent superoxide in the CNS, we tested the effect of i.c.v. administration of apocynin, an inhibitor of NADPH oxidase, or tempol, a superoxide dismutase mimetic, on AP. Intracerebroventricular administration of apocynin had no effect on the AP in either *Alk1^+/−^* or *Alk1^+/+^* mice, whereas i.c.v. administration of tempol resulted in a similar decrease in AP in both groups. These results suggest that excess superoxide anion production in the brain was not responsible for the higher AP observed in *Alk1^+/−^* mice.

The final aim of our study was to find a direct link between the deficiency in ALK-1 and the phenotype observed. It is widely known that TGF-β1 exerts a neuroprotective function, protecting cultured neurons from hypoxia and excitotoxic, apoptotic and metabolic damage (Krieglstein et al., 1995; Prehn and Krieglstein, 1994). In neurons, activation by TGF-β1 of the ALK-1/Smad1/5 signaling pathway mediates activation of the antiapoptotic nuclear factor-κB, thus contributing to neuronal survival (Konig et al., 2005). Another member of the TGF-β superfamily, BMP-9, signaling through the ALK-1/Smad1/5 pathway, is a differentiation factor for cholinergic neurons of the CNS. Intracerebroventricular injection of BMP-9 increases levels of ACh *in vivo* and in adult mice subjected to unilateral transection of the fimbria-fornix pathway, thus preventing loss of septal cholinergic neurons in the hippocampus (Lopez-Coviella et al., 2000; Schnitzler et al., 2010). In these cells, the BMP-9 signaling pathway activates the canonical ALK-1/Smad1/5 pathway, and this would be the mechanism responsible for the induction of the cholinergic phenotype in the basal forebrain (Schnitzler et al., 2010). Our data show a reduced number of cholinergic neurons and a lower ChAT-ir in anterior basal forebrain from *Alk1^+/−^* than in *Alk1^+/+^* mice. These data suggest that the deficiency in the expression of ALK-1 receptors could be altering the ability of the cholinergic neurons studied to respond appropriately to factors such as TGF-β and BMP, which have a known neurotrophic activity and promote cholinergic differentiation, maintaining the cholinergic phenotype of differentiated cells (Lopez-Coviella et al., 2000; Schnitzler et al., 2010).

Several studies show that medial septal cholinergic neurons in culture require a constant and continuous supply of nerve growth factor to maintain their integrity and normal function, whereas administration of BMP-9 has nerve growth factor-like effects, in that BMP-9 induces nerve growth factor release in septal cultures and BMP-9 is involved in crucial neurogenesis (Jonakait et al., 1998; Mazzoni and Kenigsberg, 1996). Thus, the deficiency in ALK-1 might be affecting the integrity of the basal forebrain cholinergic population, and this pathway might be the cause of the decrease in the number of ChAT-ir neurons observed in *Alk1^+/−^* compared with *Alk1^+/+^* mice. Therefore, it would be interesting to carry out studies of both ALK-1 expression and the state of cholinergic populations from caudal regions of the brain of *Alk1^+/−^* animals, in an attempt to determine the relationship that some neuronal populations in this region could have with the central cholinergic control of cardiovascular mechanisms (Kubo et al., 2002; Li et al., 2009; Varagic et al., 1991).

## MATERIALS AND METHODS

### Animals

Generation of *Alk1^+/−^* mice has been described previously (Oh et al., 2000). A breeding colony of adult *Alk1^+/−^* mice has been maintained in the pathogen-free facilities for genetically modified mice at the University of Salamanca, and backcrossed with C57Bl/6 mice for nine generations. Routine genotyping of DNA isolated from mouse tail biopsies was performed by PCR using the primers previously reported (Oh et al., 2000). Studies were performed in 3- to 5-month-old male mice.

### Mouse maintenance

Animals were kept in controlled ambient conditions (Animal Experimentation Service, University of Salamanca, Spain) in a temperature-controlled room with a 12 h-12 h light-dark cycle and were reared on standard chow (Panlab, Barcelona, Spain) and water *ad libitum*. In all procedures, mice were treated in accordance with the recommendations of the US National Institutes of Health (NIH) *Guide for the Care and Use of Laboratory Animals* and the Spanish Government. The procedure was approved by the Bioethics committee of the University of Salamanca.

### Measurement of arterial pressure, heart rate and locomotor activity

Arterial pressure (AP) and heart rate (HR) were measured by the tail-cuff method using tail-cuff equipment adapted for mice, and by radiotelemetry as previously described (Chamorro-Jorganes et al., 2010). Locomotor activity was also measured by telemetry. In some cases, the pressor response was measured by direct cannulation in anesthetized animals, as previously described (Perez-Rivero et al., 2006).

For assessment of prolonged drug effects, mice were familiarized during several weeks to restraint in a plastic restraining chamber for several minutes, in a quiet place. The AP was measured using tail-cuff equipment adapted for mice (Letica, Barcelona, Spain). The drugs tested were as follows: tempol (4-hydroxy-2,2,6,6-tetramethylpiperindine-*N*-oxyl), 1 mmol/ml p.o., captopril 100 µg/ml p.o., losartan 150 µg/ml p.o., atenolol 500 µg/ml p.o., prazosin 50 µg/ml p.o. (these drugs were obtained from Sigma-Aldrich, St Louis, MO, USA) and bosentan 250 µg/ml p.o. (Actelion Ltd, Allschwil, Switzerland). Measurements made on the 4th and 5th days after drug administration were similar and averaged for each animal. Studies were conducted in at least five *Alk1^+/+^* and five *Alk1^+/−^* mice.

To assess circadian AP changes or acute changes in AP, radiotelemetry techniques were used (Chamorro-Jorganes et al., 2010). The substances tested were as follows: acetylcholine (ACh) 1 μg/kg body weight, sodium nitroprusside (SNP) 2 mg/kg body weight, L-NAME 50 mg/kg body weight, angiotensin II (AngII) 0.30 and 0.80 mg/kg body weight, tempol 20 mg/kg body weight, losartan 10 mg/kg body weight, atenolol 5 mg/kg body weight and prazosin 5 mg/kg body weight. Effects of the different substances injected intraperitoneally were assessed in consecutive days in at least seven *Alk1^+/+^* and seven *Alk1^+/−^* mice.

In some cases, the pressor response was measured by direct cannulation in anesthetized animals (Perez-Rivero et al., 2006). For this purpose, the animals were anesthetized with a combination of ketamine 78 mg/kg body weight, diazepam 6 mg/kg body weight and atropine 0.15 mg/kg body weight, i.p. Body temperature was maintained at 37°C, and tracheotomy was performed. The right carotid artery was cannulated with PE-10 tubing and connected to a pressure transducer, and AP was continuously recorded with a digital data recorder (MacLab/4e; AD Instruments, Australia) and analyzed using Chart version 3.4. The left jugular vein was catheterized for the administration of vasoactive substances. Supplemental doses of anesthetic agents were administered as required.

### Isolated hindlimb perfusion

Isolated hindlimb perfusion experiments were also performed as previously described (Perez-Rivero et al., 2006). The aorta was cannulated with PE-50 tubing in the caudal direction near the aortic bifurcation and connected to a pressure transducer using a T-shaped tube for continuous measurement of perfusion pressure, recorded with a digital data recorder (MacLab/4e; AD Instruments). Hindlimbs were continuously perfused, using a peristaltic pump, with warmed (37°C), oxygenated Krebs-Henseleit solution (pH 7.4) containing dextran, 40 g/l, at a flow rate that produced a perfusion pressure of 80 mmHg. Hindlimb vascular resistance was calculated by dividing the perfusion pressure by the perfusion flow. AngII was administered in the perfusion fluid as previously reported (Jerkic et al., 2004).

### Vascular functionality of thoracic aorta rings

The thoracic aorta was cleaned of adherent fat, and rings 2 mm long from *Alk1^+/+^* and *Alk1^+/−^* sibling mice were cut and placed in a bath containing 5 ml Krebs solution, gassed with carbogen (95% O_2_ and 5% CO_2_) and kept at 37°C. In these experiments to measure contractility, a wire myograph was used (Multiwire Myograph System, Model 620M; DMT, Denmark), as previously described (Sauzeau et al., 2006). In relaxation experiments, aortic rings were precontracted with 3×10^−7^ M PE (with induces about 80% of the maximal contraction) before adding the relaxant agents.

### Mouse echocardiography

Transthoracic echocardiography was performed using a Vivid 7 (GE Medical Systems) cardiac ultrasound machine equipped with a 10-14 MHz transducer il3L (GE Medical Systems) by a single experienced operator who was blinded to the mouse genotype, as previously described (Arechederra et al., 2013).

### Electrocardiographic measurements in conscious mice using radiotelemetry

Telemetric electrocardiogram (ECG) recordings in conscious mice were carried out using an implantable telemetry system with ECG transmitters (ETA-F10; Data Sciences International, St Paul, MN, USA) as previously described (Arechederra et al., 2013).

### Intracerebroventricular injection

Mice were anesthetized with a combination of ketamine 78 mg/kg body weight, diazepam 6 mg/kg body weight and atropine 0.15 mg/kg body weight, i.p., and body temperature was maintained at 37°C. The right carotid artery was cannulated with PE-10 tubing connected to PE-50 tubing and to a pressure transducer for continuous measurement of AP, which was recorded with a digital data recorder (MP150, four-channel amplifier; Biopac Systems Inc.) and analyzed using AcqKnowledge 4.1 (a data acquisition and analysis program). Mice were placed in a stereotaxic apparatus, and a 33-gauge needle connected to a Hamilton microsyringe was inserted into the lateral ventricle, as previously described (Grande et al., 2011). After recording the basal AP, artificial cerebroventricular fluid (0.13 M NaCl) or tempol (10 µmol/μl in 3 µl of 0.13 M NaCl, 10 min), losartan (10 μg/μl in 3 µl of 0.13 M NaCl, 10 min), AngII (200 ng/0.5 μl in 0.13 M NaCl, 8 min) or apocynin [4.8 mg/ml (ethanol), 0.5 μl/min in 0.13 M NaCl, 18 min] was infused manually into the lateral ventricle, and changes in systolic AP and HR were recorded in real time for 30 min after injection.

### Analysis of renal function

Urine was obtained from individual mice housed in metabolic cages for 24 h. Urine was collected in graduated cylinders containing 100 μl of 0.1% sodium azide (to minimize bacterial growth) and 1 ml of mineral oil (to avoid evaporation). Blood samples (150 μl) were collected from the caudal vein of these animals to measure the creatinine concentration in plasma. Urine and plasma creatinine concentrations were determined by a modification of Jaffé's reaction method. Urine and plasma sodium concentrations were measured using automated clinical equipment.

### Nitrite measurements

The nitrite concentration was determined in plasma and urine by a modification of the Griess reaction as described previously (Valdivielso et al., 2001). Briefly, 500 μl samples were mixed with 250 μl of Griess reagent (1% sulfanilamide and 0.1% naphthyl ethylenediamine dihydrochloride, in 2.5% orthophosphoric acid; Sigma-Aldrich) and incubated for 15 min at room temperature. Absorbance was measured at 560 nm. Calibration was carried out using sodium nitrite.

### RT-PCR and western blot analysis

Total RNA was isolated using Nucleospin RNAII (Macherey-Nagel, Düren, Germany), according to the manufacturer's instructions. Quantitative RT-PCR was performed in triplicate as previously described (Munoz-Felix et al., 2014). Western blot analysis was performed basically as previously described (Rodriguez-Barbero et al., 2001). The specific primers used for PCR and the specific antibodies used for western blot are described in Tables S2 and S3, respectively.

### Enzyme-linked immunosorbent assays

Blood samples were obtained from the caudal vein of mice in tubes containing EDTA (1 mM). Blood samples were centrifuged at 1600 ***g*** for 15 min at 4°C, and plasma was collected. Plasma concentrations of AngII were determined by the AngII EIA kit (EK-002-12; Phoenix Pharmaceuticals Inc.) following the manufacturer's instructions. The plasma concentrations of mouse renin were analyzed using the ELISA Kit for renin (REN; SEA889Mu; USCNK). Epinephrine and norepinephrine plasma levels [2-CAT (A-N) Research ELISA, BA E-5400; Labor Diagnostika Nord, Nordhorn, Germany] were quantified following the supplier's instructions.

### Determination of angiotensin II-converting enzyme activity

Angiotensin II-converting enzyme activity was measured by a fluorometric enzymatic assay, using an artificial substrate of the enzyme, hippuryl-L-histidyl-L-leucine (Sigma-Aldrich). The hippuryl-L-histidyl-L-leucine substrate was prepared at 25 mM in 25 mM NaOH solution. Lung and kidney samples were homogenized in buffer containing 50 mM HEPES at pH 7.4, 25 mM ZnCl_2_, 150 mM NaCl, Triton X-100 and 0.5% protease inhibitor cocktail without EDTA (Roche). Homogenates were centrifuged for 10 min at 14,000 ***g*** and 6°C to clarify. The protein concentration was measured in the supernatants of each sample, and 1 µg of protein was mixed with the substrate solution consisting of 5 mM hippuryl-L-histidyl-L-leucine, 0.9 M NaCl and 0.4 M borate buffer at pH 8.3 to a final volume of 75 μl. The samples were incubated at 37°C for 20 min, and the reaction was stopped by adding 180 μl of 0.28 M NaOH. Subsequently, 15 μl of *o*-phthaldialdehyde (20 mg/ml in methanol) was added (Sigma-Aldrich), whose reaction product emitted fluorescence. After 10 min at room temperature, the reaction was acidified with 30 μl of 3 N HCl. Each sample was centrifuged for 5 min at 800 ***g***, and 200 μl of the supernatant was transferred to a black 96-well plate. The fluorescence was measured at an excitation wavelength of 360 nm with emission at 485 nm.

### Tissue preparation and ChAT immunohistochemistry

After induction of deep general anesthesia with an overdose of sodium pentobarbital, mice were perfused transcardially with fresh Ringer's calcium-free buffer, followed by freshly depolymerized 4% paraformaldehyde in 0.1 M phosphate buffer (pH 7.4), at room temperature. Heart and kidneys were removed, embedded in paraffin, and standard staining with hematoxylin and eosin or Masson's trichrome was performed. Brains were dissected and cryoprotected, and serial coronal sections (40 µm) were obtained. A standard immunostaining procedure was used for demonstration of neuronal ChAT in the septal area, which entailed incubating the sections in a primary polyclonal goat anti-ChAT antibody (catalog no. AB144P; Millipore; 1:1000 in Tris-buffered saline containing 0.2% Triton X-100 and 1% fetal calf serum) for 72 h at 4°C. The sections were then washed and incubated for 90 min in the corresponding biotinylated secondary antibody according the recommendations of the manufacturer (Vector Laboratories, Burlingame, CA, USA). After removal of the secondary antibodies, the sections were washed and incubated for a further 90 min in Avidin/Biotin complex according to the manufacturer's instructions (Vector Laboratories) and developed using 3,3′-diaminobenzidine as the chromogen. All sections were mounted on slides, dehydrated, and coverslipped using Entellan Neu (Merck, Darmstadt, Germany). In addition, the histological sections of a paraffin block from each animal were counterstained with the Nissl staining for cytoarchitectonic references.

### Statistical analyses

The non-parametric Mann-Whitney *U*-test was used for comparisons among the different groups. When data were paired, they were analyzed using Student's *t*-test. When there were several groups and/or several treatments, data were analyzed by two-way ANOVA followed by Newman-Keuls test. All our data are expressed as the mean±s.e.m. Statistical significance was set at *P<*0.05. For all experiments, at least five animals were used per group. Statistical analyses were performed using the Statgraphics software package (Manugistics Inc., Herndorn, VA, USA).
